# Ginsenoside F1 Promotes Cytotoxic Activity of NK Cells via Insulin-Like Growth Factor-1-Dependent Mechanism

**DOI:** 10.3389/fimmu.2018.02785

**Published:** 2018-11-28

**Authors:** Hyung-Joon Kwon, Heejae Lee, Go-Eun Choi, Soon Jae Kwon, Ah Young Song, So Jeong Kim, Woo Seon Choi, Sang-Hyun Hwang, Sun Chang Kim, Hun Sik Kim

**Affiliations:** ^1^Department of Biomedical Sciences, University of Ulsan College of Medicine, Asan Medical Center, Seoul, South Korea; ^2^Department of Clinical Laboratory Science, Catholic University of Pusan, Busan, South Korea; ^3^Department of Laboratory Medicine, Asan Medical Center, University of Ulsan College of Medicine, Seoul, South Korea; ^4^Department of Biological Sciences, Korea Advanced Institute of Science and Technology, Daejeon, South Korea; ^5^Department of Microbiology, University of Ulsan College of Medicine, Asan Medical Center, Seoul, South Korea

**Keywords:** natural killer cell, ginsenoside, cytotoxicity, cancer surveillance, IGF-1

## Abstract

Ginsenosides are the principal active components of ginseng and are considered attractive candidates for combination cancer therapy because they can kill tumors and have favorable safety profiles. However, the overall benefit of ginsenosides remains unclear, particularly in cancer immunosurveillance, considering the controversial results showing repression or promotion of immune responses. Here we identify a potentiating role of ginsenoside F1 (G-F1) in cancer surveillance by natural killer (NK) cells. Among 15 different ginsenosides, G-F1 most potently enhanced NK cell cytotoxicity in response to diverse activating receptors and cancer cells. G-F1 also improved cancer surveillance in mouse models of lymphoma clearance and metastatic melanoma that rely on NK cell activity. G-F1-treated NK cells exhibited elevated cytotoxic potential such as upregulation of cytotoxic mediators and of activation signals upon stimulation. NK cell potentiation by G-F1 was antagonized by insulin-like growth factor (IGF)-1 blockade and recapitulated by IGF-1 treatment, suggesting the involvement of IGF-1. Thus, our results suggest that G-F1 enhances NK cell function and may have chemotherapeutic potential in NK cell-based immunotherapy. We anticipate our results to be a starting point for further comprehensive studies of ginsenosides in the immune cells mediating cancer surveillance and the development of putative therapeutics.

## Introduction

Ginseng, the root of *Panax ginseng* C. A. Meyer, has been a core component of traditional herbal medicine, especially in China and Korea, owing to the belief that it is a tonic and panacea (1–3). Currently, it is among the most widely used herbal remedies for various disorders worldwide. The pharmacological properties of ginseng are considered to be mainly attributable to ginsenosides, which are triterpene saponins consisting of a steroidal backbone with sugar moieties (4, 5). Ginsenosides have a variety of biomedical efficacies including anti-aging, anti-diabetic, anti-cancer, and immune modulatory effects (2, 4–6). Ginsenosides differ from each other in the position, number, and type of sugar moieties, and such diversity is believed to underlie their diverse therapeutic potentials (4, 5, 7).

Ginsenosides have gained considerable attention as promising adjunct and supportive agents in the prevention and treatment of cancer based on their favorable efficacy and safety profiles (2, 5). In addition, they have been shown to augment the anti-cancer effects of conventional chemotherapeutic agents (8, 9). These studies focused on elucidating the anti-cancer effects of ginsenosides in the context of their interaction with cancer cells. Multiple mechanisms of action for ginsenosides have been proposed for such anti-cancer effects, including the induction of apoptosis and growth arrest and the inhibition of angiogenesis and metastasis (5, 10, 11). Despite studies suggesting diverse therapeutic potential against cancer cells, the overall benefit of ginsenosides in cancer chemoprevention and therapy remains unclear, particularly in cancer immunosurveillance (3). Conflicting studies have revealed that ginsenosides either repress or promote immune responses (6, 12–14), thereby contributing to maintaining the homeostasis of the immune system. Accordingly, investigating the effect of different ginsenosides on the function of immune cells mediating anti-cancer responses is relevant, considering crucial role of immune system in cancer surveillance.

Natural killer (NK) cells are considered key effectors in cancer immunosurveillance and a promising component of cancer therapeutics owing to their intrinsic selectivity against cancer cells (15–17). Unlike T and B cells, NK cells are in the “ready-to-kill” status and, thereby, provide early protection against cancer cells without the requirement for prior sensitization and major histocompatibility complex (MHC)-restriction (18, 19). NK cells have an array of innate receptors that respond to cellular transformation and, thereby, can trigger effector functions following the recognition of cancerous cells via direct cytolysis and production of cytokines (e.g., interferon [IFN]-γ) and chemokines [e.g., macrophage inflammatory protein (MIP)-1α]. They also contribute indirectly to anti-cancer immunity by regulating the activation of antigen-presenting cells and T cells. Numerous human studies have shown that NK cell functional deficiency is a key risk factor for developing various types of cancer and is a typical feature of diverse patients with cancer (20–22). Moreover, the extent of NK cell dysfunction correlates with the cancer prognosis (23, 24). In support of this notion, high incidences of tumors and metastasis were reported in experimental mice with defects in NK cell number, function, or both (15, 25, 26). This correlation has encouraged relentless interest and efforts for developing strategies that promote NK cell reactivity against cancer cells safely and efficaciously.

Previous *in vitro* studies have shown that treatment with the ginsenoside fraction significantly enhanced NK cell cytotoxicity of mouse splenocytes and human peripheral blood mononuclear cells (PBMCs) (27–29). Ginsenosides that effectively enhance NK cell effector function include ginsenoside Rg1 (G-Rg1) (28, 29). Splenocytes from G-Rg1-treated mice showed an enhanced natural killing activity (28). G-Rg1 but not G-Rb1, G-Re, G-Rc, and G-Rd also moderately enhanced natural cytotoxicity and antibody-dependent cellular cytotoxicity (ADCC) of human PBMCs (29). These studies were performed with a mixed population of immune cells and, therefore, the direct effects of ginsenosides on NK cells have not yet been investigated. Ginsenosides also undergo metabolic transformations in the body, such as stepwise deglycosylation of sugar moieties, resulting in different or adverse functional outcomes (4, 30, 31). Furthermore, the mechanism of NK cell potentiation by ginsenosides remains unexplored. In this study, we reveal that G-F1, a deglycosylated metabolite of G-Rg1, potentiates NK cell cytotoxicity in a direct and insulin-like growth factor 1 (IGF-1)-dependent mechanism, thus providing a clue for the mysterious efficacy of ginsenosides in NK cell activation.

## Materials and methods

### Ginsenosides

Standard grade ginsenosides, including compound K (C-K), F2, protopanaxadiol (PPD), Rb1, Rb2, Rc, Rd, Rg3, Rh2, F1, PPT, Re, Rg1, Rg2, and Rh1 were purchased from Nanjing Zelang Medical Technology Co., Ltd. (China) or were obtained by enzymatic conversion as previously described (32). A cherry-picking test for assessing NK cell degranulation was performed with ginsenosides purchased from LKT Labs Co., Ltd. (China). The purity of all ginsenosides used was > 98%.

### Cell isolation and culture

Human blood samples from normal healthy donors were drawn for research purposes under a protocol approved by the institutional review board with informed consent. PBMCs were isolated by density gradient centrifugation using lymphocyte separation medium (MP Biomedicals, Solon, OH). Human NK cells were purified from PBMCs by negative selection using a human NK cell isolation kit (StemCell Technologies) as described (33). These cells were 97–99% CD3-CD56+ as assessed by flow cytometry. The human NK cell line NKL was cultured in RPMI 1640 supplemented with 10% fetal bovine serum (FBS), 1 mM sodium pyruvate, and 200 U/ml recombinant IL-2 (rIL-2) (Roche). K562, 721.221, and KCL-22 cells were cultured in Iscove's modified Dulbecco's medium (IMDM) supplemented with 10% FBS and 2 mM L-glutamine. YAC-1, RMA, and RMA-s cells were cultured in RPMI1640 supplemented with 5% FBS and 2 mM L-glutamine. B16F10 cells were cultured in Dulbecco's modified Eagle's medium (DMEM) supplemented with 10% FBS and 2 mM L-glutamine. In some experiments, PBMCs were activated with 200 U/ml rIL-2 for 24 h. Male C57BL/6 mice were used at 6–7 weeks old. Murine NK cells were purified from splenocytes by negative selection using a mouse NK cell isolation kit (StemCell Technologies) according to the manufacturer's instructions.

### NK cell expansion

Primary human NK cells were expanded from normal donors as previously described (34), with some modifications. PBMCs (1.5 × 10^6^ cells) were incubated in a 24-well tissue culture plate with 100 Gy-irradiated K562-mb15-41BBL cells (a gift of D. Campana, National University of Singapore; 1 × 10^6^ cells) in stem cell growth medium (SCGM; CellGenix) supplemented with 10% FBS and 10 U/mL rIL-2. The medium was changed every 2 days and replaced with fresh medium with rIL-2. After 1 week of coculture, residual T cells were depleted with a CD3+ selection kit (StemCell Technologies). Purified NK cells were further expanded for 2 additional weeks in SCGM supplemented with 10% FBS, 100 U/mL rIL-2, and 5 ng/mL rIL-15 (PeproTech). The resulting cell populations were 96–99% CD3–CD56+, as assessed using flow cytometry.

### Antibodies and reagents

Antibodies for assessing NK cell receptors and signaling molecules related to NK cell activation were obtained from the following sources: NKG2D (149810; R&D Systems); CD244/2B4 (C1.7; Beckman Coulter); isotype control mouse IgG1 (MOPC-21; Sigma-Aldrich); pS473 Akt (9271), Akt (9272), pY202/204 Erk1/2 (9101), and Erk1/2 (9102) (Cell Signaling Signaling). Goat F(ab′)2 anti-mouse IgG was obtained from Jackson ImmunoResearch. The fluorochrome-conjugated antibodies were used in the flow cytometric analyses: anti-human CD3-PerCP (SK7), anti-human CD16-PE (3G8), anti-human CD56-R-phycoerythrin (PE) (NCAM16.2), anti-human CD107a-FITC (H4A3), anti-human IFN-γ-FITC (25723.11), anti-human DNAM-1-PE (DX11), anti-human NKp46-PE (9E2), anti-mouse CD3ε-PerCP (145-2C11), anti-mouse NKp46-PE (29A1.4), anti-mouse CD107a-FITC (1D4B), anti-mouse H-2Kb-PE (AF6-88.5), and anti-mouse CD16/CD32 (mouse Fc Block; 2.4G2) from BD Biosciences (San Jose, CA); anti-human 2B4-PE (C1.7) anti-human KIR2DL1/S1-PE (CD158a; EB6) from Beckman Coulter; anti-human NKG2D-PE (149810) from R&D Systems; anti-human perforin-Alexa 647 (dG9) and anti-human granzyme B-Alexa 647 (GB11) from BioLegend. Anti-human IGF-1 antibody (antigen-affinity-purified polyclonal goat IgG) and normal goat IgG control were obtained from R&D Systems. Ionomycin, JB1, and human IGF-1 were purchased from Sigma-Aldrich and CFSE from Molecular Probes. IGF-1 release into the supernatants was determined by human IGF-1 Quantikine ELISA (R&D Systems).

### CD107a degranulation assay

NK cell degranulation was assessed by determining the cell surface expression of CD107a as described (34). For stimulation, IL-2-activated PBMCs or primary expanded NK cells were stimulated with an equal number of K562, 721.221, KC-22, or P815 cells preincubated with monoclonal antibodies specific for activating receptors. Murine NK cells isolated from splenocytes were stimulated with YAC-1 target cells for 4 h. Lymphocytes were gated on forward scatter/side scatter, and the CD107a expression of CD3-CD56+ human NK cells and CD3-NKp46+ mouse NK cells was analyzed by flow cytometry.

### Intracellular cytokine assay

Cytokine production by NK cells was assessed by determining the intracellular expression of IFN-γ as described (34). IL-2-activated PBMCs were stimulated with an equal number of K562 cells for 1 h at 37°C. Thereafter, brefeldin A (GolgiPlug; BD Biosciences) and monensin (GolgiStop; BD Biosciences) were added and the cells were incubated for an additional 5 h, making a total incubation time of 6 h. Lymphocytes were gated on forward scatter/side scatter, and the intracellular IFN-γ expression of CD3-CD56+ human NK cells was analyzed by flow cytometry.

### NK cell cytotoxicity assay

The cytotoxicity of NK cells against sensitive target cells was assessed using a europium-based cytotoxicity assay as described previously (35). Primary NK cells and NKL cells served as effector cells.

### *In vivo* lymphoma clearance assay

Lymphoma cells expressing MHC class-I (RMA) were labeled with a low CFSE concentration (1 μM), whereas those with defective expression of MHC class-I (RMA-s) were labeled with a high CFSE concentration (4 μM). The cells were mixed in a 1:1 ratio (1 × 10^6^ cells per each cell type) and injected i.p. into C57BL/6 mice. To deplete the NK cells, i.p. injections of rabbit anti-asialo-GM1 (10 μl; Cedarlane Laboratories Ltd) or control rabbit serum (Sigma-Aldrich) were performed for 3 consecutive days before an injection of CFSE-stained RMA and RMA-s cells at a ratio of 1:1. Rejection of NK cell-sensitive RMA-s relative to NK cell-resistant RMA cells in the peritoneal cavity was measured using flow cytometry and calculated as follows: 1 – ([CFSE^low^/CFSE^high^]input/[CFSE^low^/CFSE^high^]output) × 100%.

### *In vivo* pulmonary metastasis assay

Cultured B16F10 cells were recovered by Detachin (Gelantis) treatment and then injected into the tail veins of C57BL/6 mice (2 × 10^5^ cells/200 μl DPBS or 5 × 10^5^ cells/200 μl DPBS). After 14 day of tumor implantation, the mice were euthanized and the lungs were fixed in Fekete's solution. The number of nodules of metastatic tumor cells in the lungs was determined by using a dissection microscope (Nikon). To deplete the NK cells, mice were administered i.p. injections of 10 μL rabbit anti-asialo-GM1 (Cedarlane Laboratories Ltd.) while the control mice were injected with rabbit serum (Sigma-Aldrich). The injections of anti-asialo-GM1 or rabbit serum were performed 1 day before an i.v. injection of B16F10 cells, and then twice a week administration until euthanasia.

### Measurement of intracellular Ca^2+^

Expanded primary NK cells were loaded with fluo-4 AM (Invitrogen; 2 μg/mL) in HBSS supplemented with 1% FBS and probenecid (Sigma-Aldrich; 4 mM) for 30 min at 37°C. The cells were washed, plated onto poly-L-lysine-coated coverslips, and then transferred to a recording chamber (Live Cell Instruments) placed on an inverted confocal microscope (Carl Zeiss LSM710). Changes in intracellular Ca^2+^ flux were measured over time following stimulation through NKG2D and 2B4 and then Ca^2+^ ionophore ionomycin (4 μM) as positive control. Intracellular Ca^2+^ flux was also measured by flow cytometry in cells labeled with 2 μg/mL fluo-4 AM as described (35).

### Perforin and granzyme B staining

Expanded primary NK cells (2 × 10^5^ cells) were incubated with the indicated ginsenosides or IGF-1 for 20 h at 37°C. Cells were then surface-stained with anti–CD3-PerCP and anti–CD56-PE monoclonal antibodies. After washing the cells twice with flow cytometry buffer, they were incubated in BD Cytofix/Cytoperm solution. The cells were washed twice with BD Perm/Wash buffer before and after staining with anti–perforin-Alexa647 or anti-granzyme B-Alexa 647 monoclonal antibody. Thereafter, the cells were analyzed by flow cytometry gated on CD3-CD56+ NK cells.

### Real-time PCR analysis

The *PRF1* and *GzmB* expression levels in human NK cells were assessed using a Real-Time PCR analysis as described previously (34). After isolation of total RNA using the RNeasy kit (Qiagen, Hilden, Germany), cDNA was synthesized from 1 μg RNA using the ReverTra Ace qPCR RT kit (Toyobo, Osaka, Japan) according to manufacturer protocols. Real-time PCR amplification and analysis were conducted using the SYBR Green Real-Time PCR Master Mix (Toyobo) and LightCycler 480 Real-Time PCR system (Roche Diagnostics). The PCR conditions were as follows: preheating for 10 min at 95°C, 40 cycles of 95°C (30 s), 60°C (30 s) and 72°C (30 s). Melting curve analysis was done using the default settings of the device. The β-actin mRNA level was used as a normalization control. The following PCR primers were used: 5′-CGCCTACCTCAGGCTTATCTC-3′ (forward) and 5′-CCTCGACAGTCAGGCAGTC-3′ (reverse) for *PRF1*; 5′-CCCTGGGAAAACACTCACACA-3′ (forward) and 5′-CACAACTCAATGGTACTGTCGT-3′ (reverse) for *GzmB*; 5′-AGGAAGTACATTTGAAGAACGCAAGT-3′ (forward) and 5′-CCTGCGGTGGCATGTCA-3′ (reverse) for *IGF-1*; 5′-ACTCCATCATGAAGTGTGACG-3′ (forward) and 5′-CATACTCCTGCTTGCTGATCC-3′ (reverse) for *ACTB*.

### Annexin V-FITC/PI staining

To measure cell death, NK cells and target cells (K562 or 721.221) were treated with or without G-Rg1 or G-F1 for 5 or 24 h and then analyzed using a FACS Canto instrument and the annexin V-FITC apoptosis detection kit from BD Biosciences.

### Statistical analysis

Each graph was generated from at least three independent experiments. Statistical analyses were conducted by two-tailed Student's *t*-test or one-way ANOVA using the GraphPad Prism 5.0. software. Dunnett's test and Newman-Keuls's test were performed for multiple comparison post-tests. *P* < 0.05 were considered statistically significant.

## Results

### Ginsenoside F1 promotes the natural cytotoxicity and IFN-γ production of NK cells

**Figure 1:**
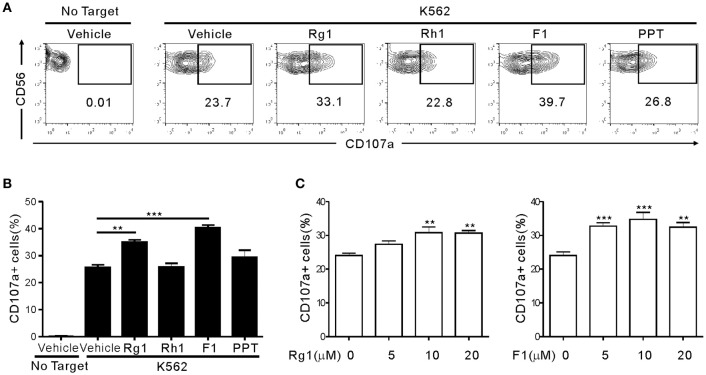
Ginsenoside F1 (G-F1) enhances the natural cytotoxicity of NK cells. PBMCs exposed to IL-2 were pretreated with the indicated ginsenosides (10 μM) for 20 h and were then mixed with K562 cells in the presence of ginsenosides. After 2 h incubation at 37°C, cytotoxic degranulation of NK cells was measured by cell surface expression of CD107a on CD3-CD56+ NK cells. **(A,B)** Representative flow cytometry profiles **(A)** and summary graphs of statistical bar charts **(B)** showing percentage of CD107a+ NK cells in four independent experiments. **(C)** Bar charts showing expression of CD107a by NK cells at varying ginsenoside concentrations. Data are expressed as means ± s.e.m. ^**^P < 0.01; ^***^P < 0.001 by one-way ANOVA with the Dunnett's test.

To determine whether ginsenosides affect NK cell function, we first screened 15 different ginsenosides including G-Rg1 by assessing their effect on cytotoxic degranulation of NK cells against K562 target cells (Figure S1). The degranulation efficiency correlates with target cell lysis by NK cells and is indicated by CD107a positivity on NK cells (36). PBMCs were isolated from healthy donors, activated with interleukin (IL)-2 and then mixed with K562 cells for 2 h after preincubation with 10 μM ginsenosides for 20 h. NK cells were identified as the CD3-CD56+ population among size-gated lymphocyte population. As expected, G-Rg1 augmented the degranulation of NK cells against K562 cells (Figure S1A), and this increase was statistically significant (Figure S1B). These analyses also revealed that six other ginsenosides (G-F2, G-Rb1, G-Rg3, G-Rh2, G-F1, and G-Rg2) have the potential to enhance the degranulation of NK cells. Then, the seven ginsenosides obtained from different commercial source were subjected to a cherry-picking test, which revealed that G-Rg3, G-F1, and G-Rg1 consistently and significantly increased the degranulation of NK cells, and G-F1 showed the most potent effect (Figure S2). In comparison, the increase in NK cell degranulation was not statistically significant with other four ginsenosides. Thus, we focused our study on G-F1, a deglycosylated metabolite of G-Rg1. Of note, among the G-Rg1 metabolites (Figure S3), G-F1 enhanced the degranulation of NK cells most potently, and its effect was better than that of G-Rg1 (Figures 1A,B). In comparison, G-Rh1 and protopanaxatriol (PPT) did not significantly affect NK cell degranulation. Exposure to an increasing dose of both ginsenosides (5–20 μM) caused a dose-dependent and significant increase in NK cell degranulation; the effect was most pronounced at 10 μM G-F1 and also potent at 5 μM G-F1 (Figure 1C).

Next, we assessed the effect of G-F1 and G-Rg1 on NK cell proinflammatory cytokine production. The intracellular expression of IFN-γ was assessed in CD3-CD56+ NK cells in response to K562 cells. As observed with cytotoxic degranulation, IFN-γ production of NK cells was significantly enhanced by treatment with G-F1 and G-Rg1 (10 μM), and the effects of G-F1 were more potent (Figure S4). Although the degree to which the ginsenosides increased NK cell functions varied by donor, a reproducible enhancement of NK cell functions by G-F1 and G-Rg1 was observed. Collectively, these results suggest that G-Rg1 and its metabolite G-F1 promote the natural cytotoxicity and IFN-γ production of NK cells. Furthermore, the NK cell effector function was most potently enhanced by G-F1 treatment.

### Ginsenoside F1 promotes the cytolytic activity of highly pure NK cells

**Figure 2:**
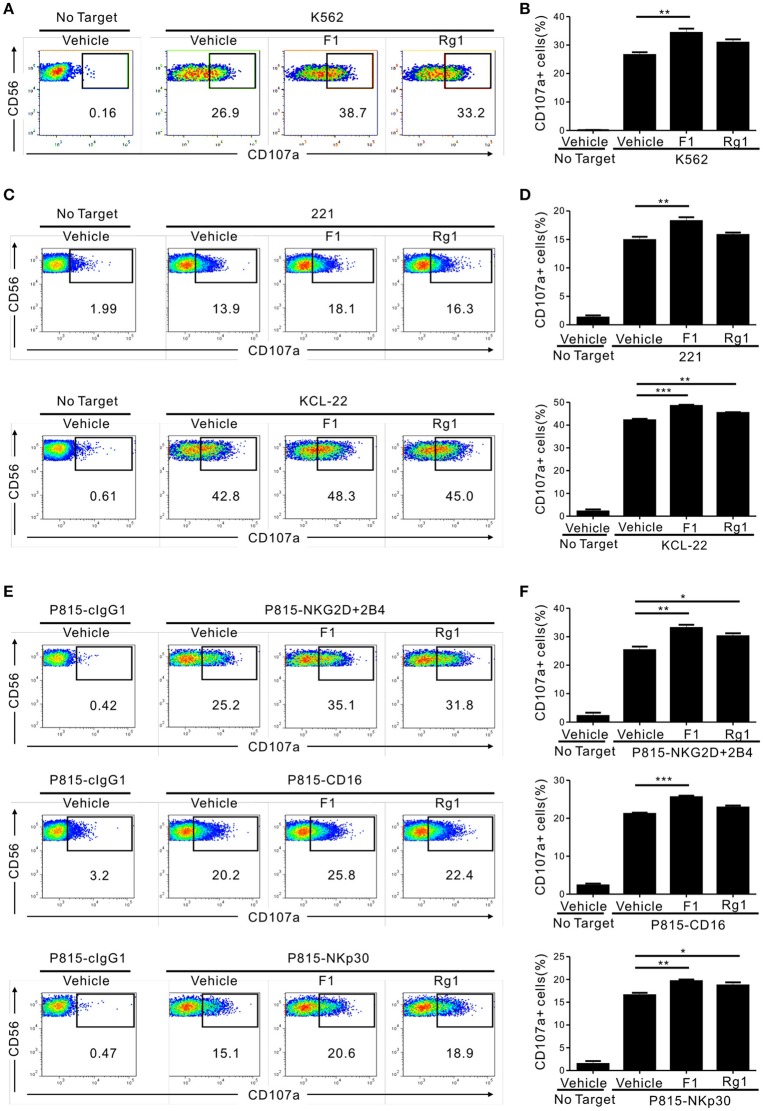
Ginsenoside F1 (G-F1) promotes the cytotoxicity of NK cells through diverse activating receptors. **(A–D)** Expanded primary NK cells were pretreated with the indicated ginsenosides (10 μM) for 20 h and were then incubated with different target cells such as K562 cells **(A,B)** 721.221 cells or KCL-22 cells **(C,D)** in the presence of ginsenosides and fluorochrome-conjugated anti-CD107a monoclonal antibody. After incubation for 2 h, cells were stained with fluorochrome-conjugated monoclonal antibody to CD56, and the level of CD56+CD107a+ NK cells was measured using flow cytometry. Representative results **(A,C)** and summary graphs of statistical bar charts **(B,D)** showing percentage of CD107a+ NK cells in at least three independent experiments. **(E,F)** Expanded primary NK cells were pretreated with the indicated ginsenosides (10 μM) for 20 h and then mixed with P815 cells preincubated with isotype control monoclonal antibody (cIgG1) or monoclonal antibodies to the indicated receptors in the presence of ginsenosides and fluorochrome-conjugated anti-CD107a monoclonal antibody. After incubation for 2 h, cells were analyzed using flow cytometry as described in **(A–D)**. Representative result **(E)** and summary graph of statistical bar charts **(F)** showing percentage of CD107a+ NK cells in four independent experiments. Data are expressed as means ± s.e.m. ^*^p < 0.05; ^**^p < 0.01; ^***^p < 0.001 by one-way ANOVA with the Dunnett's test.

We then determined whether G-F1 enhances NK cell function directly on highly pure NK cells or indirectly via G-F1-responsive non-NK cells in PBMCs. To this end, highly pure primary NK cells expanded with feeder cells and cytokines were used as the effector cells. NK cell degranulation was assessed using three different target cell lines that express heterologous ligands for different activating receptors on NK cells. As observed in the PBMCs, exposure of primary expanded NK cells to G-F1 (10 μM) caused a significant augmentation of NK cell degranulation against K562 cells; the effect was more potent than that of G-Rg1 (Figures 2A,B). As expected, G-Rh1 and PPT did not affect the degranulation of NK cells (Figure S5). Likewise, a similar increase in NK cell degranulation by G-F1 (10 μM) was observed after stimulation with different target cells [i.e., 721.221 (hereafter referred to as 221) and KCL-22] (Figures 2C,D). In support, NK cell cytotoxicity against K562 and KCL-22 cells was also enhanced by G-F1 (10 μM) treatment (Figure S6A).

Next, we investigated whether the enhancement of NK cell function by G-F1 also occurred in NK cell activation through defined and diverse activating receptors. NK cell effector functions can be triggered by different activating receptors such as immunoreceptor tyrosine-based activation motif (ITAM)-coupled (e.g., CD16 and NKp30) and non-ITAM-coupled receptors (e.g., NKG2D and 2B4). To this end, we used P815 target cells that enable specific receptor-dependent NK cell stimulation. P815 cells are FcR+ and, upon incubation with monoclonal antibodies specific to NK cell-activating receptors (for example, NKG2D and 2B4), bind the antibodies via the Fc-region. In doing so, they can activate NK cells via specific activating receptors in a direct cell–cell contact (34). Stimulation of primary expanded NK cells via combination of NKG2D and 2B4, CD16 and NKp30 induced an apparent degranulation, which was significantly enhanced following treatment with G-F1 (10 μM) but not consistently G-Rg1 (Figures 2E,F). The observation seen in primary NK cells was also applicable to the NK cell line, as G-F1 (10 μM) treatment augmented the cytotoxicity of NKL cells against 221 cells and in response to co-engagement of NKG2D and 2B4 (Figure S6B). G-F1 and G-Rg1 at up to 40 μM did not significantly affect the viability of target cells tested, as assessed by annexin-V/ propidium iodide (PI) staining, thus ruling out their direct cytotoxic effect on target cells. Collectively, these results suggest that G-F1 enhances NK cell cytotoxic function triggered by different activating receptors.

### Ginsenoside F1 enhances the cytotoxicity of NK cells *in vivo*

The ability of NK cells to kill abnormal cells lacking MHC class-I molecules *in vivo* is well-established (37). To determine whether G-F1 enhances NK cell function *in vivo*, we used a syngeneic tumor clearance model. Specifically, mouse lymphoma cells expressing MHC class-I (RMA) and those with defective expression of MHC class-I (RMA-s) were co-injected into mice pretreated with G-F1. In this model, NK cells preferentially kill MHC class-I-deficient RMA-s cells over their parent RMA cells (38). We confirmed defective MHC class-I expression in RMA-s cells compared to RMA cells (Figure S7A). To facilitate their identification, RMA and RMA-s cells were labeled with different concentrations of carboxyfluorescein succinimidyl ester (CFSE), and then equal numbers of cells were co-injected intraperitoneally (i.p.) into the mice (Figure S7B). As expected, we observed a selective clearance of RMA-s cells over RMA cells 12 h post-injection (Figures S7C,D). Elimination of RMA-s cells was mainly mediated by NK cells, as it was prevented by NK cell depletion with an antibody specific to the ganglioside asialo-GM1 (Figures S8C,D). The efficacy of anti-asialo-GM1-mediated depletion of NK cells was confirmed in the splenocytes of mice stained to detect CD3ε**-**NKp46+ NK cells (Figures S8A,B).

**Figure 3:**
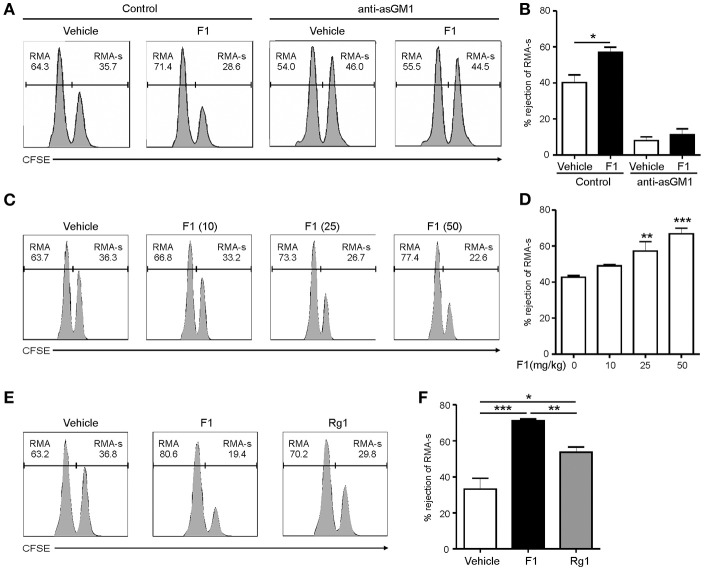
Ginsenoside F1 (G-F1) enhances NK cell-mediated lymphoma clearance *in vivo*. **(A,B)** C57BL/6 mice (*n* = 4 per group) received either G-F1 (25 mg/kg; i.p.) or vehicle with or without anti-asialo-GM1 (10 μL) for 3 consecutive days before an i.p. injection of CFSE-stained RMA (low CFSE concentration) and RMA-s cells (high CFSE concentration) at a ratio of 1:1. After 6 h post challenge of RMA:RMA-s cells, NK cell-sensitive RMA-s cells relative to NK cell-resistant RMA cells remained in the peritoneal cavity were recovered and assessed using flow cytometry. Representative result **(A)** and summary graph of statistical bar charts **(B)** showing percentage of rejected RMA-s cells. Values represent means ± s.e.m. **(C,D)** C57BL/6 mice (*n* = 4 per group) received increasing doses of G-F1 (i.p.) for 3 consecutive days before an i.p. injection of CFSE-stained RMA and RMA-s cells at a ratio of 1:1. After 6 h post challenge, rejection of RMA-s cells over RMA cells was analyzed using flow cytometry as described in **(A,B)**. Representative result **(C)** and summary graph of statistical bar charts **(D)** showing percentage of rejected RMA-s cells. Values represent means ± s.d. **(E,F)** C57BL/6 mice (*n* = 5 per group) received the indicated ginsenosides (25 mg/kg; i.p.) for 3 consecutive days before an i.p. injection of 1:1 mix of CFSE-stained RMA and RMA-s cells. After 6 h post challenge, rejection of RMA-s cells over RMA cells was analyzed as described in **(A,B)**. Representative result **(E)** and summary graph of statistical bar charts **(F)** showing percentage of rejected RMA-s cells. Values represent means ± s.e.m. ^*^p < 0.05; ^**^p < 0.01; ^***^p < 0.001 by Student's *t*-test **(B)**, one-way ANOVA with the Dunnett's test **(D)**, and one-way ANOVA with the Neuman-Keuls's test **(F)**.

Next, to study the effect of G-F1 on the clearance of RMA-s cells, the experiment was repeated via administration of recipient mice with G-F1 prior to NK cell-sensitive RMA-s cell injection. Pretreatment with 25 mg/kg G-F1 (i.p.) significantly enhanced the peritoneal clearance of RMA-s cells 6 h post challenge of RMA:RMA-s cells, which was diminished after anti-asialo-GM1-mediated depletion of NK cells (Figures 3A,B). This result indicated that G-F1 treatment most likely affect NK cells for the clearance of RMA-s cells. The increase of RMA-s cell clearance with G-F1 treatment was dose-dependent at up to 50 mg/kg and statistically significant at 25–50 mg/kg (Figures 3C,D). As observed with the *in vitro* results, the capacity to eliminate NK cell-sensitive RMA-s cells was significantly enhanced by treatment with G-F1 and G-Rg1 (25 mg/kg), with a more pronounced effect by G-F1 (Figures 3E,F). These results suggest that administration of G-F1 enhances cancer surveillance *in vivo* in a lymphoma clearance model that relies on NK cell activity.

**Figure 4:**
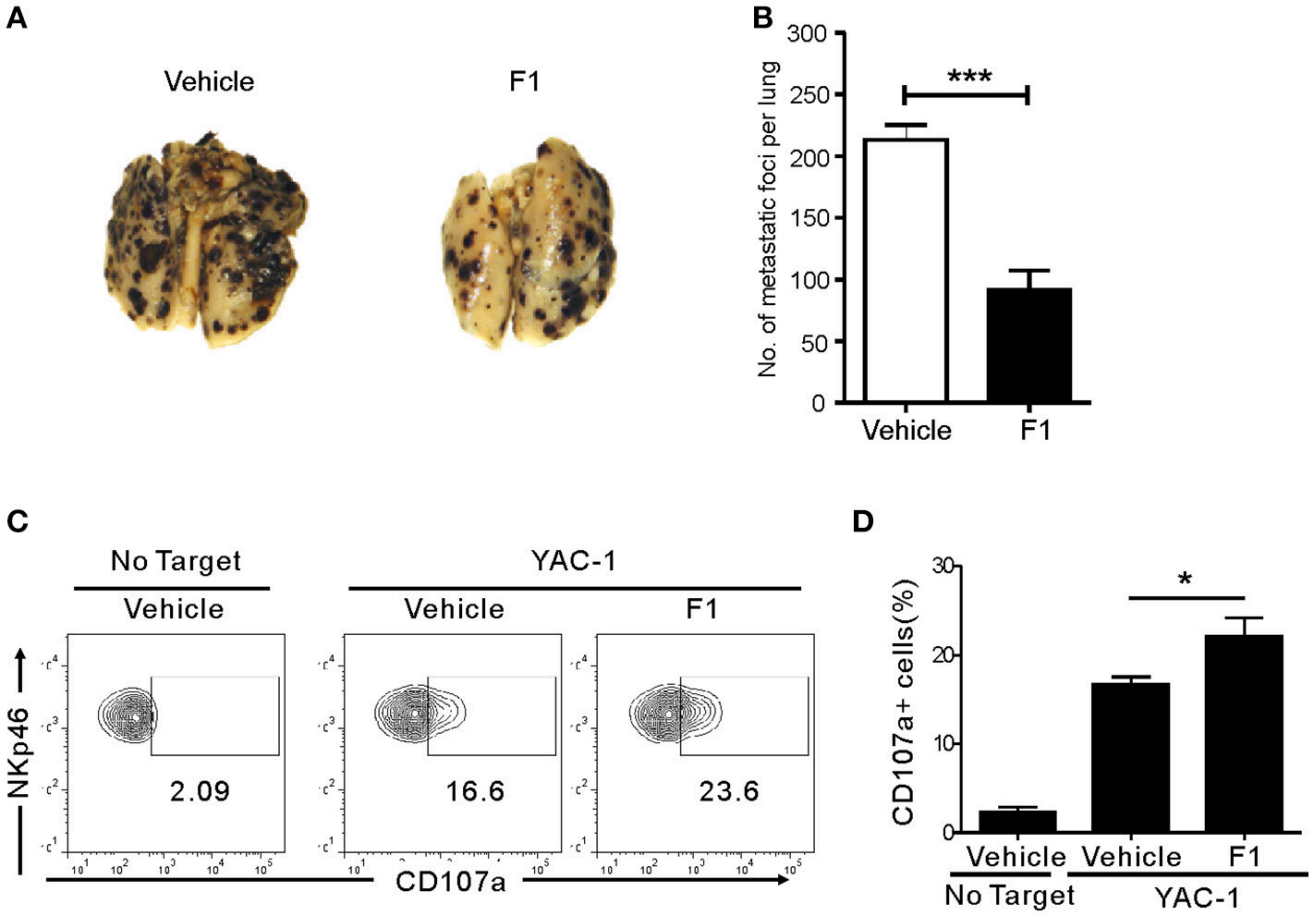
Ginsenoside F1 (G-F1) enhances NK cell-mediated protection against pulmonary metastatic melanoma *in vivo*. **(A,B)** C57BL/6 mice (*n* = 7 per group) received either G-F1 (50 mg/kg; i.p.) or vehicle for 3 consecutive days before an i.v. injection of 5 × 10^5^ B16F10 cells and thrice a week administration thereafter. After 14 day of tumor implantation, mice were euthanized and number of metastatic tumor colonies in the lungs was counted after fixation. Representative lung image **(A)** and summary graph of statistical bar charts **(B)** showing pulmonary metastases of B16F10 cells. Values represent means ± s.e.m. **(C,D)** C57BL/6 mice (*n* = 4 per group) received either G-F1 (50 mg/kg; i.p.) or vehicle as described above. Thereafter, splenic NK cells were isolated and incubated with YAC-1 target cells in a 1:1 ratio for 4 h. Cytotoxic degranulation was measured by determining surface expression of CD107a on CD3ε-NKp46+ NK cells. Representative flow cytometry profile **(C)** and summary graph of statistical bar charts **(D)** showing percentage of CD107a+ NK cells. Values represent means ± s.d. ^*^p < 0.05; ^***^p < 0.001 by Student's *t*-test.

We next studied whether G-F1 also enhances NK cell-mediated systemic antitumor effects *in vivo* in a site other than where G-F1 was administered. To this end, we used a syngeneic mouse model of experimental tumor metastasis in which B16F10 melanoma cells were injected into mice intravenously (i.v.) and assessed the effect of G-F1 treatment on pulmonary metastatic growth. B16F10 melanoma cells are highly metastatic and deficient in MHC class-I surface expression (39). The i.v. injection of B16F10 cells led to the establishment of lung metastases 2 weeks after inoculation (Figure S9). Consistent with previous reports (40, 41), systemic metastasis of B16F10 cells was mainly controlled by NK cells, as demonstrated by the substantial increase in lung metastases after depletion of NK cells by an anti-asialo-GM1 antibody (Figure S9). As expected, G-F1 significantly and effectively prevented the lung colonization of systemically injected B16F10 cells following i.p. injection of 50 mg/kg (Figures 4A,B). To assess NK cell function *ex vivo* in these tumor-bearing mice, spleen NK cells were isolated from each group of mice and incubated with YAC-1 target cells for 4 h. Compared with the control group, the G-F1-treated group showed a significant increase in cytotoxic degranulation of spleen NK cells against YAC-1 cells (Figures 4C,D). Thus, these results suggest that G-F1-mediated increased surveillance against B16F10 cells is associated with systemic enhancement of the cytotoxic potential of individual NK cells.

### Ginsenoside F1 enhances cytotoxic potential of NK cells

**Figure 5:**
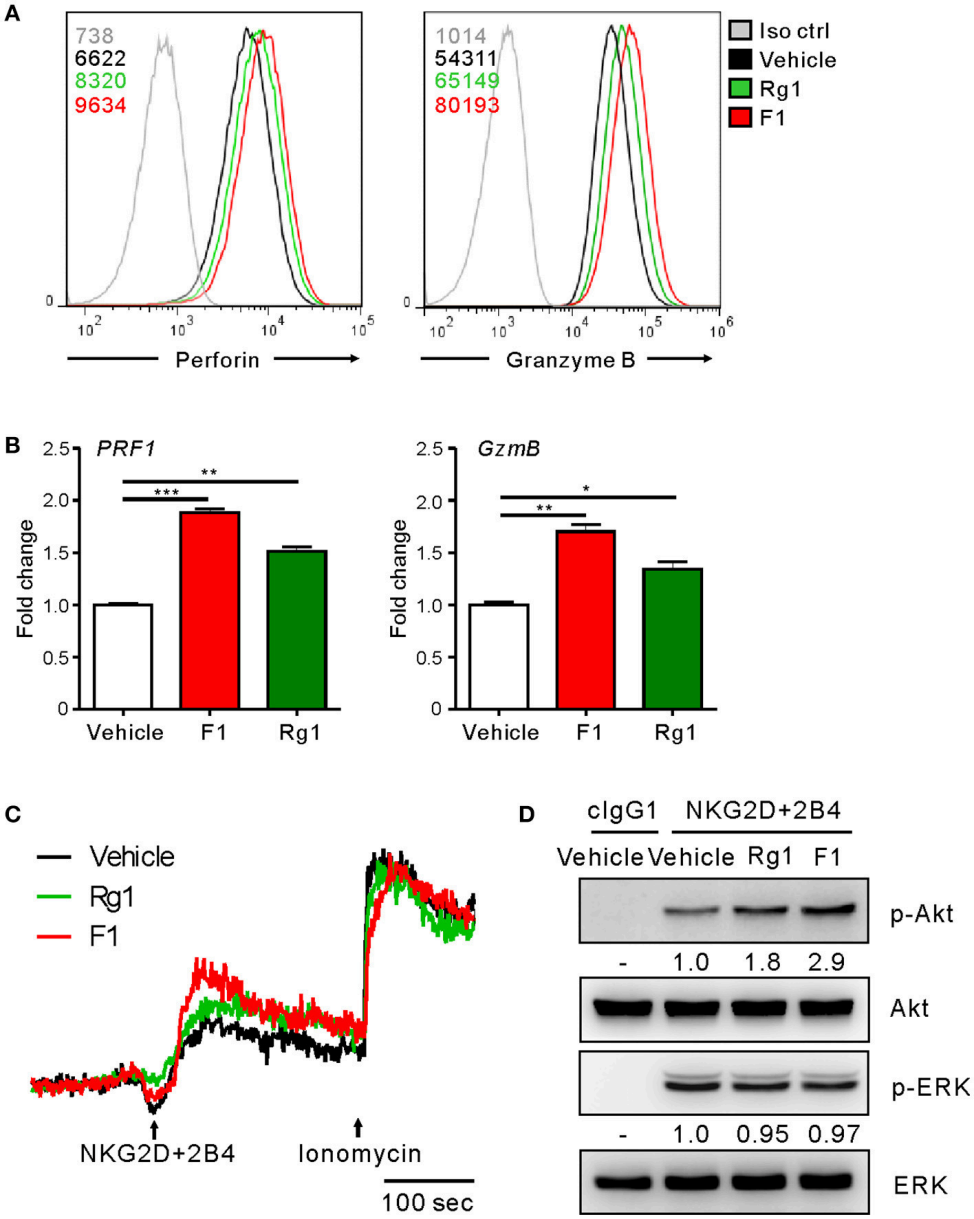
Ginsenoside F1 (G-F1) enhances the cytotoxic potential of NK cells. **(A)** Representative flow cytometry profile showing the expression (median fluorescence intensity) of the perforin (*PRF1, left panel*) and granzyme B (*GzmB, right panel*) in expanded primary NK cells after treatment with the indicated ginsenosides (10 μM) (green or red solid line) or vehicle only (black solid line) for 20 h. Isotype control staining is shown as gray solid line. **(B)** Expanded primary NK cells were treated with the indicated ginsenosides (10 μM) for 20 h. Relative mRNA levels of *PRF1* (*left panel*) and *GzmB* (*right panel*) were determined by quantitative real-time PCR and normalized to actin mRNA. Data are presented as fold change relative to vehicle-treated cells. Values represent means ± s.d. **(C)** Ca^2+^ mobilization in expanded primary NK cells treated with the indicated ginsenosides (10 μM) for 20 h. NK cells were stimulated through NKG2D and 2B4 (indicated by the arrow) after the measurement of baseline Ca^2+^ concentrations for 30 s. Ionomycin was treated sequentially and used as positive control. Changes in fluorescence are shown as a function of time. **(D)** Expanded primary NK cells were treated with the indicated ginsenosides (10 μM) for 20 h and stimulated with NKG2D and 2B4 by receptor crosslinking for 2 min. Cell lysates were immunoblotted with Abs to phospho-Akt at serine 473 (pS473), Akt, phospho-Erk1/2, or Erk1/2. The normalized intensities of the phosphorylated Akt and ERK relative to their total forms were quantified using Image J and are presented. ^*^p < 0.05; ^**^p < 0.01; ^***^p < 0.001 by Student's *t*-test. Data are representative of at least three independent experiments.

To gain an insight into the mechanism of action of G-F1 on NK cells, we first investigated the expression of NK activating and inhibitory receptors, including NKG2D, 2B4, and CD16, through which G-F1 enhanced NK cell cytotoxicity. The expression of NK cell-receptors examined was virtually unaffected on NK cells following G-F1 (10 μM) treatment (Figure S10). These results suggest that G-F1-mediated potentiation of NK cell cytotoxicity is not primarily linked to altered expression of NK activating receptors. In comparison, the expression levels of perforin and granzyme B, effector molecules mediating target cell killing following NK cell degranulation, were apparently increased in NK cells by G-F1 (10 μM) treatment (Figure 5A), as determined by flow-cytometry analysis. This effect was more potent than that of G-Rg1, which correlated with the increase in NK cell cytotoxicity. Furthermore, similar and significant upregulation of mRNA expression of genes encoding perforin (*PRF1*) and granzyme B (*GzmB*) was noted in NK cells treated with these ginsenosides (Figure 5B). These results indicate that G-F1 enhances the cytotoxic potential of NK cells by increasing the levels of cytotoxic effector molecules.

We then investigated the intracellular signaling mechanisms of G-F1-stimulated NK cells. As calcium-mediated signaling pathway is critical to NK cell functions upon target cell recognition (33, 42, 43), we assessed the effect of G-F1 on calcium mobilization triggered by the NK activating receptor. Stimulation of primary NK cells through co-engagement of NKG2D and 2B4 receptors evoked a clear increase in intracellular Ca^2+^ in NK cells, which was further increased modestly by G-F1 (10 μM) compared with the vehicle control (Figure 5C). In comparison, NK cells irrespective of ginsenoside treatment showed a similar degree of calcium mobilization upon stimulation with Ca^2+^ ionophore ionomycin. Besides phospholipase C (PLC)-γ-calcium pathway, signaling molecules linked to cytotoxic degranulation of NK cells include phosphoinositide 3-kinase (PI3K)-Akt and the mitogen-associated protein kinase (MAPK), extracellular signal-regulated kinase (Erk) (44, 45). Compared with the vehicle control, G-F1 (10 μM) enhanced the phosphorylation of Akt, which is downstream of PI3K activation, rather than that of Erk, induced by co-engagement of NKG2D and 2B4. Furthermore, the effect of G-F1 was more potent than that of G-Rg1 (Figure 5D). Taken together, these results suggest that G-F1 enhanced the cytotoxic potential of NK cells likely through mechanisms involving upregulation of cytotoxic mediators and signaling downstream of activating receptors.

### Ginsenoside F1 enhances NK cell cytotoxicity via an IGF-1 pathway

**Figure 6:**
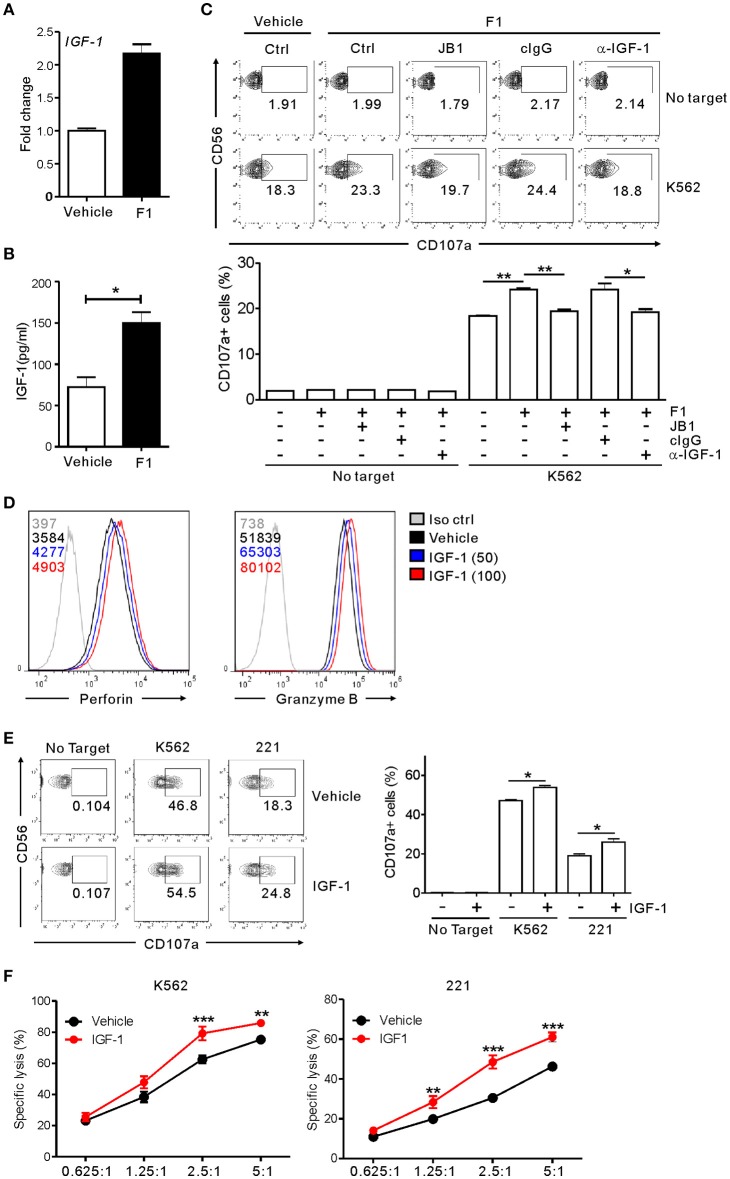
NK cell potentiation by ginsenoside F1 is associated with IGF-1. **(A)** *IGF-1* expression in expanded primary NK cells treated with either G-F1 (10 μM) or vehicle, as assessed by quantitative real-time PCR at 6 h of culture. **(B)** IGF-1 levels in the supernatant of expanded primary NK cells treated with either G-F1 (10 μM) or vehicle, as assessed by ELISA at 12 h of culture. **(C)** PBMCs exposed to IL-2 were pretreated with G-F1 (10 μM) for 20 h in the presence of either IGF-1-neutralizing antibody (α-IGF-1) or JB1 for blockade of IGF-1 pathway. Thereafter, cytotoxic degranulation of NK cells was assessed after incubation with K562 cells as in Figure 1. Representative flow cytometry profile (*upper panel*) and summary graph of statistical bar charts *(lower panel*) showing percentage of CD107a+ NK cells. **(D)** Representative flow cytometry profile showing the expression (median fluorescence intensity) of the perforin (*left panel*) and granzyme B (*right panel*) in primary expanded NK cells after treatment with increasing doses of IGF-1 (50–100 ng/mL; blue or red solid line) or vehicle only (black solid line) for 20 h. Isotype control staining is shown as gray solid line. **(E)** Expanded primary NK cells were pretreated with IGF-1 (100 ng/mL) or vehicle for 20 h and were incubated with K562 and 221 cells for 2 h in the presence of fluorochrome-conjugated anti-CD107a monoclonal antibody. Cells were then stained with fluorochrome-conjugated monoclonal antibody to CD56, and the level of CD56+CD107a+ NK cells was measured using flow cytometry. Representative results (*left panel*) and summary graphs of statistical bar charts (*right panel*) showing percentage of CD107a+ NK cells. **(F)** Lysis of target cells (K562 or 721.221) by expanded primary NK cells after treatment with IGF-1 (100 ng/mL) or vehicle only for 20 h at the indicated effector to target (E:T) cell ratio. Cytotoxicity against target cells was measured after 2 h with the Europium assay. Error bars represent the s.d. ^*^p < 0.05; ^**^p < 0.01; ^***^p < 0.001 by Student's *t*-test **(B,C,E)** and two-way ANOVA with the Bonferroni's test **(F)**. Data are representative of at least three independent experiments.

Having observed the enhancement of NK cell cytotoxicity by G-F1 and its metabolic precursor G-Rg1, we hypothesized that G-F1 and G-Rg1 share a common mechanistic feature of NK cell activation. G-Rg1 triggers activation of the IGF-1 receptor (IGF-1R) pathway (46, 47) that promotes the cytotoxic activity of human NK cells (48, 49). Considering that G-F1 is a deglycosylated metabolite of G-Rg1, we investigated the involvement of IGF-1 in G-F1-mediated NK cell potentiation. We observed that treatment with G-F1 (10 μM) triggers an upregulation in the mRNA expression and subsequent production of IGF-1 by highly pure human NK cells (Figures 6A,B), corroborating the previous study of the ability of human NK cells to produce IGF-1 (50). Furthermore, the involvement of IGF-1 in NK cell potentiation was assessed using an IGF-1-neutralizing antibody and JB1, an IGF-1 peptide analog that functions as an IGF-1 receptor antagonist by competing with IGF-1 (47). Simultaneous exposure to the IGF-1-neutralizing antibody or JB1 blocked the G-F1-induced enhancement of NK cell degranulation after stimulation with K562 cells (Figure 6C), indicating the dependence of G-F1-mediated NK cell potentiation on IGF-1 pathway. Finally, we evaluated whether IGF-1 directly modulates cytotoxic potential of NK cells. We observed that exposure of NK cells to IGF-1 (50–100 ng/mL) caused a dose-dependent increase in the expression levels of perforin and granzyme B (Figure 6D). Consistent with this enhanced cytotoxic potential of NK cells, treatment of NK cells with IGF-1 significantly increased the degranulation (Figure 6E) and cytotoxicity of NK cells (Figure 6F) against K562 and 221 cells. Taken together, our results suggest that IGF-1, which is upregulated by G-F1 treatment, may be an important contributing factor in the GF-1-induced enhancement of the cytotoxic potential of NK cells.

## Discussion

Ginsenosides, which are the major active ingredients of ginseng, have gained increasing attention as potential adjunctive or supportive agents in cancer therapy, largely due to their anti-cancer, anti-fatigue, and immune–stimulatory properties (3, 5, 6). However, numerous studies have demonstrated that ginsenosides also exhibit immune-suppressive effects on cultured immune cells and in animals (6, 13, 14). Accumulating evidence suggests that this dual effect of ginsenosides on immune cells is a consequence of their diverse chemical structures and likely involves different sugar moieties (4, 30). Although considered important to maintain homeostasis of the immune system, such diversity in structure and function of ginsenosides remains a major challenge for therapeutic development (5). In this study, we present evidence to support that among 15 different ginsenosides, G-F1, a deglycosylated metabolite of G-Rg1, significantly potentiates NK cell functions, such as cytotoxicity and IFN-γ production, and thereby enhances the susceptibility of diverse cancer cells to NK cells. We found that G-F1 promoted NK cell cytotoxicity via diverse activating receptors such as both ITAM- and non-ITAM-coupled receptors, thus accounting for its potentiation of NK cell cytotoxicity against diverse cancer cells expressing heterogeneous ligands for NK activating receptors. The chemopreventive potential of G-F1 was supported by its significant NK cell-dependent inhibition of tumor burden in different *in vivo* models of syngeneic lymphoma clearance and pulmonary metastatic melanoma. NK cells are major effectors of cancer immunosurveillance owing to their intrinsic capacity to rapidly kill cells undergoing carcinogenesis. NK cell dysfunction often leads to the development of various types of cancer and poor prognosis (15, 24). Accordingly, these findings raise an intriguing possibility that the potentiation of endogenous NK cells or *in vitro* expanded NK cells for adoptive therapy hold promise in cancer treatment. In this respect, our results suggest that G-F1 is eligible for consideration as a promising candidate for NK cell-based cancer immunotherapy. In support, we observed that G-F1 at up to 50 mg/kg did not cause any toxic and lethal effect in *in vivo* study, thus suggesting favorable efficacy in safe dosages; however, systematic studies testing diverse dose ranges would be required to establish the effect of G-F1 on NK cells in the context of various preclinical and clinical settings.

In this study, we also observed that G-Rg1 treatment augmented NK cell cytotoxicity against various target cells, consistent with previous studies of G-Rg1-mediated enhancement of natural killing activity (28, 29). In addition, we found that G-Rg1 affected NK cell cytotoxicity via multiple activation pathways, similar to the results obtained with G-F1. Of note, the effect of G-F1 on NK cell effector functions was more potent than that of G-Rg1. The given ginsenosides can be transformed by stepwise cleavage of sugar moieties through the process of steaming or metabolic digestion in the intestinal tract, which results in the formation of new type of ginsenosides (4, 30, 31). G-Rg1 is mainly converted to G-Rh1 and G-F1, and then to the aglycone PPT via stepwise deglycosylation. As the deglycosylation process of ginsenoside is considered crucial to its pharmacological activity (30), our results of higher potency of G-F1 than G-Rg1 support the view of ginsenoside as a “prodrug” that is metabolized to the active form via deglycosylation (51). In general, ginsenoside metabolites can be absorbed more readily from the intestinal tract than the parent compounds because of their increased hydrophobicity after deglycosylation, with similar or different pharmacological actions (31, 52). Supporting this notion, two degradation products of the G-Rg1 or G-Re, namely G-Rh1 and G-F1, were shown to likely reach the systemic circulation in humans after oral administration and thereby suggested as pharmacologically active ginsenosides (31). Among the G-Rg1 metabolites, G-F1 but not G-Rh1 and PPT significantly enhanced NK cell cytotoxicity, which was consistent using both PBMCs and highly pure primary NK cells. These results suggest a structure-dependent effect of ginsenosides on NK cell effector function. G-Rg1 is composed of two glucose moieties attached to the α-OH at C-6 and β-OH at C-20 of the dammarane backbone (4). In comparison, G-F1 and G-Rh1 are monoglucosylated ginsenosides that have a glucose residue at C-20 and C-6, respectively, and PPT is the aglycone without a sugar moiety. Based on our results, we speculated that, in the PPT group, the sugar moiety attached at the C-20 is required, whereas that at the C-6 is dispensable or inhibitory, for promoting NK cell effector functions. Considering the dependence of the anti-cancer activities of different ginsenosides on the number and position of sugar moieties (4, 5), further studies would be required to establish the structure-function relationship of ginsenosides encompassing diverse structural moieties with respect to their direct NK stimulatory potential.

A mechanistic study indicated that the G-F1-potentiated NK cell cytotoxicity was not associated with altered expression levels of activating receptors but could be attributed to an upregulation of cytotoxic mediators and activation signals triggered by NK activating receptors. This may account for the conserved potentiating effect of G-F1 on human and mouse NK cells despite some clear differences in NK cell receptors between men and mice (53, 54). In support of the increased cytotoxic potential of NK cells, the mRNA expression and protein levels of perforin and granzyme B, the major components of cytotoxic granules, were clearly upregulated in G-F1-treated NK cells. Moreover, G-F1 treatment led to discernible increases in Ca^2+^ mobilization and activating phosphorylation of Akt, rather than Erk, in NK cells upon stimulation with activating receptors among the signaling pathways required for cytotoxic degranulation. Considering the selectivity of the signaling pathways affected, G-F1 may target selective signaling pathways directly or indirectly by releasing certain mediators.

Further experiments revealed the involvement of the IGF-1 pathway in the potentiation of NK cell cytotoxicity by G-F1. IGF-1 was initially discovered as one of the main endocrine mediators of normal development, growth, and metabolism (55). However, it has recently been recognized as an important regulator of immune responses in both innate and adaptive immune cells (56). IGF-1 can regulate the quality and amplitude of immune responses mediated by T cells, B cells, and monocytes through its interaction with IGF-1R. Corroborating the immune regulatory function of IGF-1, IGF-1R is widely expressed on various types of immune cells including T and B lymphocytes, monocyte-macrophages, and NK cells (56, 57). Recent studies also revealed an important role of IGF-1 in NK cell development and function (48, 49, 58). IGF-1 is produced by and promotes the cytotoxic activity of human NK cells (49, 50) and the expression level of IGF-1 in NK cells correlates with the cytolytic activity of NK cells from patients who have miscarried (49) and those with hepatocellular carcinoma (58). The IGF-1R pathway is activated by G-Rg1, and there is some structural similarity between G-Rg1 and G-F1; therefore, we investigated the involvement of IGF-1 in G-F1-mediated NK cell potentiation. In this study, we demonstrated that G-F1 promoted NK cell cytotoxicity by an IGF-1-dependent mechanism by using IGF-1 measurement, a neutralizing antibody, and an antagonist. Supporting this notion, IGF-1 treatment could recapitulate the effects of G-F1-mediated upregulation of cytotoxic mediators and NK cytotoxicity, suggesting IGF-1 as a probable potentiating factor in G-F1-treated NK cells. However, the involvement of other mediator(s) and regulatory mechanisms cannot be excluded. Accordingly, further study would be required to clearly understand the mechanism of action of G-F1 on NK cells and potential involvement of IGF-1 in human cancers.

In summary, our results demonstrate that G-F1 has a previously unrecognized potential to promote NK cell effector functions. Moreover, the finding of an involvement of IGF-1 in such context provides new insight into the mechanism of action of ginsenosides on NK cells. In support of structure-dependent pharmacological effect of ginsenosides, G-F1 was more potent than G-Rg1 and its metabolites in terms of NK cell potentiation. Although caution would be required for considering G-F1 as an anti-cancer agent due to metabolic transformation, our study may contribute to developing effective strategies that harness the chemopreventive and chemotherapeutic potential of ginsenosides, particularly G-F1, in the prevention and treatment of cancer.

## Ethics statement

Human study in this work was approved by the Institutional Review Board of Asan Medical Center and all participants provided written informed consent. All animal experiments were approved by Institutional Animal Care and Use Committee (IACUC) of Asan Institute for Life Sciences and done in accordance with the approved guidelines set forth by IACUC.

## Author contributions

H-JK and HL designed and carried out most of the experiments and analyzed data. G-EC, SJaK, and SJeK performed *in vivo* experiments and analyzed data. A-YS and WC performed *in vitro* experiments and analyzed data. S-HH provided reagents and blood samples with informed consent, respectively, and helped design studies with blood samples. SK and HK conceived of the study, designed experiments, analyzed the data and wrote the manuscript, with input from all coauthors.

### Conflict of interest statement

The authors declare that the research was conducted in the absence of any commercial or financial relationships that could be construed as a potential conflict of interest.
