# Supplementary material for: Ginsenoside F1 Promotes Cytotoxic Activity of NK Cells via Insulin-Like Growth Factor-1-Dependent Mechanism
Source: Front Immunol. 2018 Nov 28;9:2785. doi: 10.3389/fimmu.2018.02785 (PMC6279892; doi:10.3389/fimmu.2018.02785)
Supplement: Supplementary file 1 [file Presentation_1.pdf]

Supplementary Material

Ginsenoside F1 promotes cytotoxic activity of NK cells via insulin-like growth factor-1-dependent mechanism

Running title: NK cell potentiation by ginsenoside F1

Hyung-Joon Kwon[1,#], Heejae Lee[1,#], Go-Eun Choi[1,3], Soon Jae Kwon[1], Ah-Young Song[1], So Jeong Kim[1], Woo Sun Choi[1], Sang-Hyun Hwang[4], Sun Chang Kim[5,*], and Hun Sik Kim[1,2,*]

[1]Department of Biomedical Sciences, [2]Department of Microbiology, University of Ulsan College of Medicine, Asan Medical Center, Seoul, Korea

[3]Department of Clinical Laboratory Science, Catholic University of Pusan, Busan, Korea

[4]Department of Laboratory Medicine, Asan Medical Center, University of Ulsan College of Medicine, Seoul, Korea

[5]Department of Biological Sciences, Korea Advanced Institute of Science and Technology (KAIST), Daejeon, Korea

[#]These authors contributed equally to this work

*Correspondence: Hun Sik Kim, E-mail: hunkim@amc.seoul.kr; and Sun Chang Kim, E-mail: sunkim@kaist.ac.kr

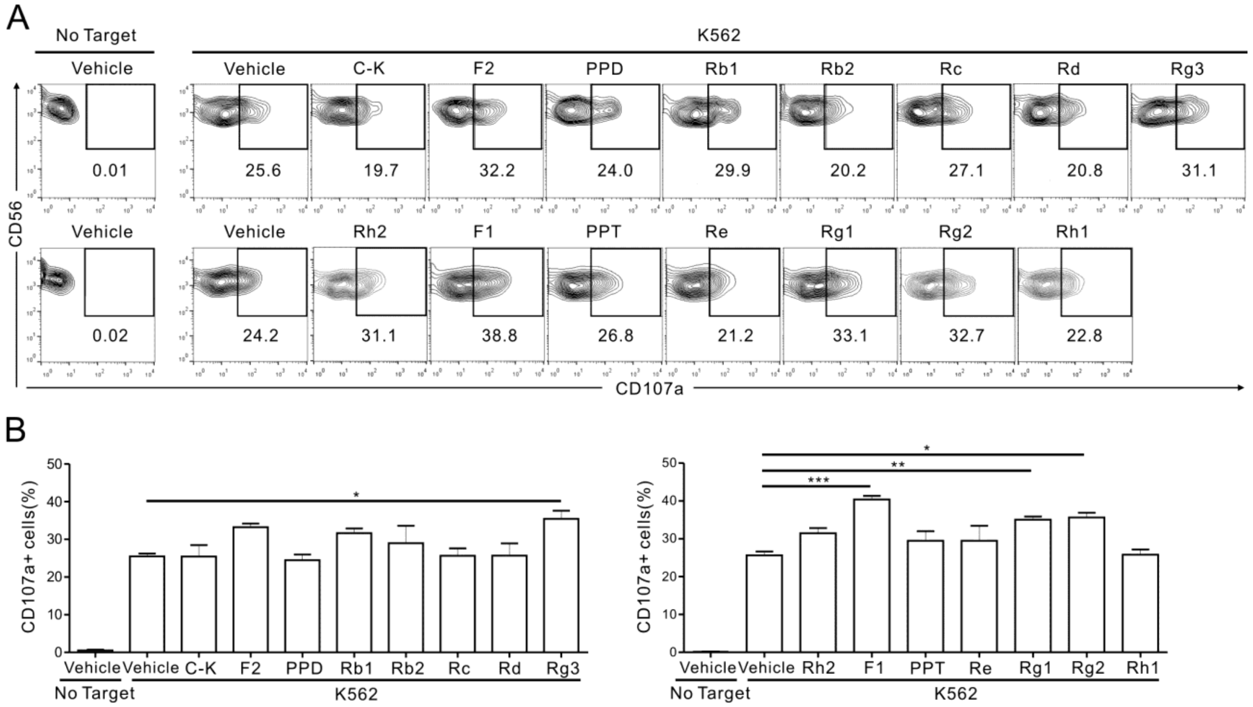

Figure S1. The effect of different ginsenosides on the natural cytotoxicity of NK cells. PBMCs exposed to IL-2 were pretreated with the indicated ginsenosides (10 μM) for 20 h and were then mixed with K562 target cells in the presence of the ginsenosides. After 2 h incubation at 37°C, cytotoxic degranulation of NK cells was determined by cell surface expression of CD107a on CD3-CD56+ NK cells. (A, B) Representative flow cytometry profiles (A) and summary graphs of statistical bar charts (B) showing percentage of CD107a+ NK cells in four independent experiments. Data are expressed as means ± s.e.m. *$P < 0.05$; **$P < 0.01$; ***$P < 0.001$ by one-way ANOVA with the Dunnett's test.

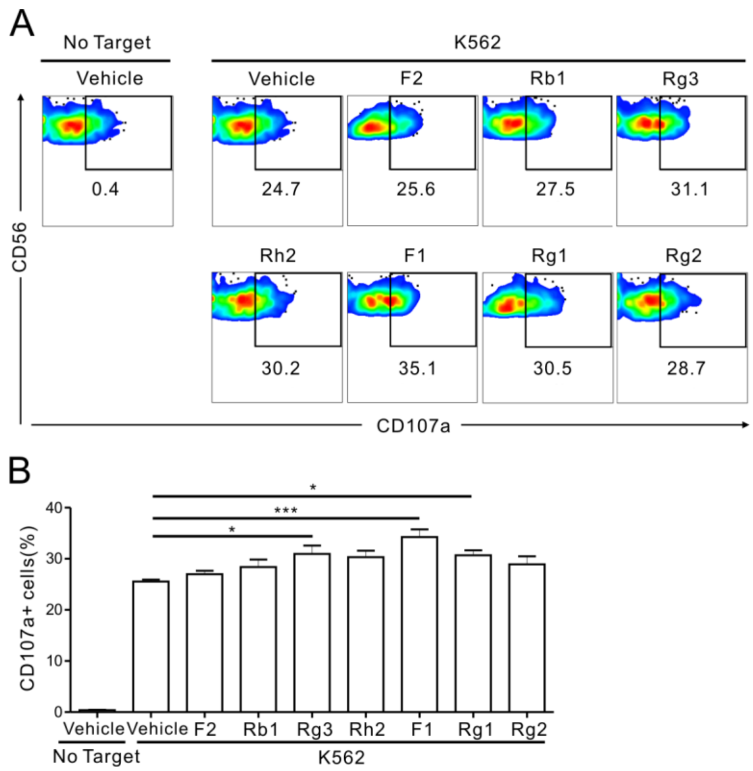

Figure S2. Ginsenoside F1 most potently enhances the natural cytotoxicity of NK cells. PBMCs exposed to IL-2 were pretreated with the indicated ginsenosides (10 μM) obtained from commercial source for 20 h and were then mixed with K562 target cells in the presence of the ginsenosides. After 2 h incubation at 37°C, cytotoxic degranulation of NK cells was determined by cell surface expression of CD107a on CD3-CD56+ NK cells. (A, B) Representative flow cytometry profiles (A) and summary graphs of statistical bar charts (B) showing percentage of CD107a+ NK cells in five independent experiments. Data are expressed as means ± s.e.m. *$P < 0.05$; ***$P < 0.001$ by one-way ANOVA with the Dunnett's test.

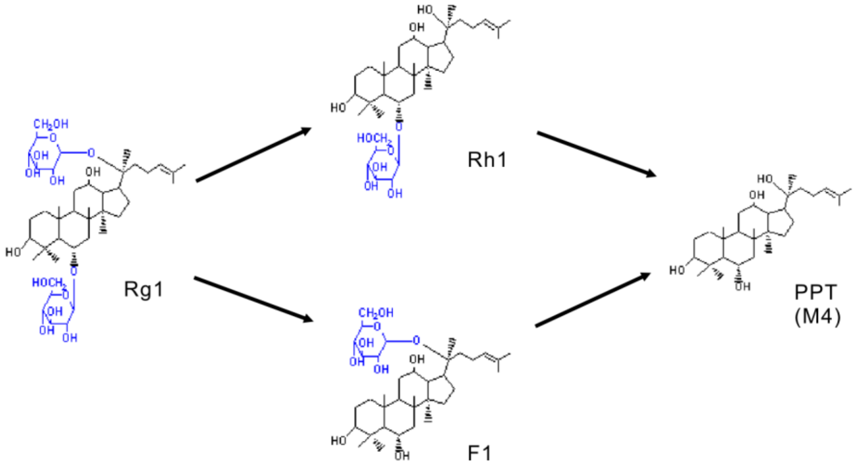

Figure S3. Putative metabolic transformation pathway of ginsenoside Rg1 in the body.

Ginsenoside Rg1 (G-Rg1) is mainly converted to monoglucosylated G-Rh1 and G-F1, and then to the aglycone PPT via stepwise deglycosylation. G-Rg1 is composed of two glucose residues attached to the α-OH at C-6 and β-OH at C-20 of the dammarane backbone. In comparison, G-Rh1 and G-F1 are monoglucosylated ginsenosides with a glucose residue at C-6 and at C-20, respectively.

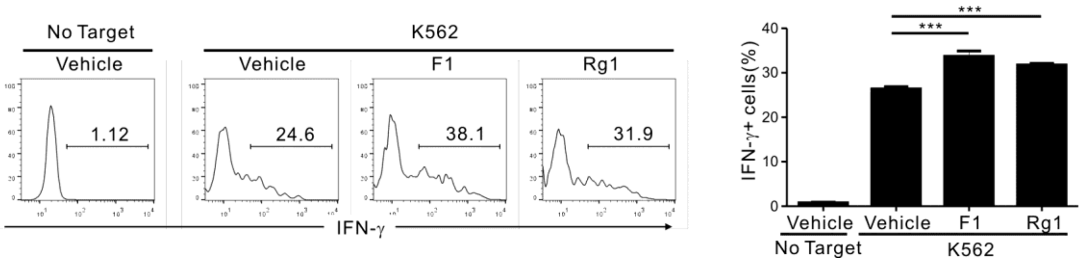

Figure S4. Ginsenoside F1 enhances IFN-γ production by NK cells.

PBMCs exposed to IL-2 were pretreated with the indicated ginsenosides (10 μM) for 20 h and were then mixed with K562 cells in the presence of ginsenosides for intracellular cytokine assay. After 6 h incubation at 37°C, cells were stained with fluorochrome-conjugated anti-CD3 and anti-CD56 monoclonal antibodies for surface staining. IFN-γ production by NK cells was measured in CD3−CD56+cells by flow cytometry after intracellular staining of IFN-γ. Representative flow cytometry profile (*left panel*) and summary graph of statistical bar charts (*right panel*) showing expression of IFN-γ by NK cells in four independent experiments. Data are expressed as means ± s.e.m. ***$P < 0.001$ by one-way ANOVA with the Dunnett's test.

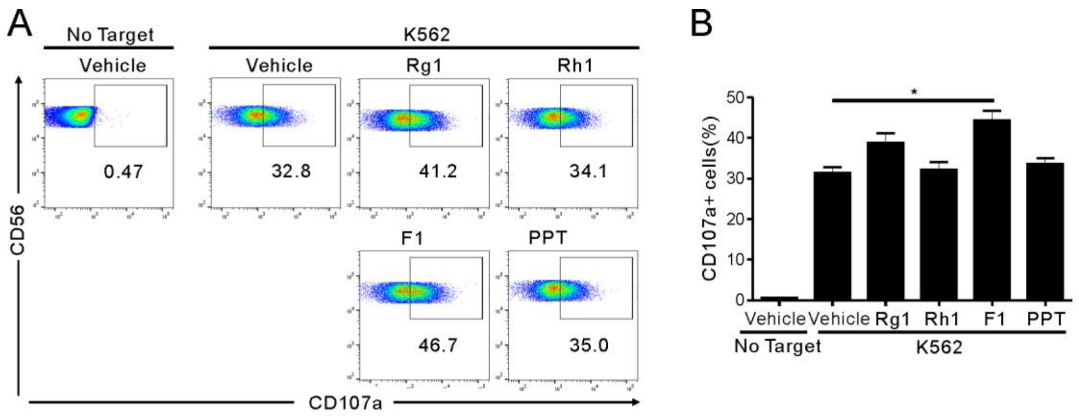

Figure S5. Ginsenoside F1 enhances the natural cytotoxicity of highly pure primary NK cells.

(A, B) Expanded primary NK cells were pretreated with the indicated ginsenosides (10 μM) for 20 h and were mixed with K562 cells for 2 h in the presence of ginsenosides and fluorochrome-conjugated anti-CD107a monoclonal antibody. Cells were then stained with fluorochrome-conjugated monoclonal antibody to CD56, and the level of CD56+CD107a+ NK cells was measured using flow cytometry. Representative results (A) and summary graphs of statistical bar charts (B) showing percentage of CD107a+ NK cells in two independent experiments. Data are expressed as means ± s.e.m. *$P < 0.05$ by one-way ANOVA with the Dunnett's test.

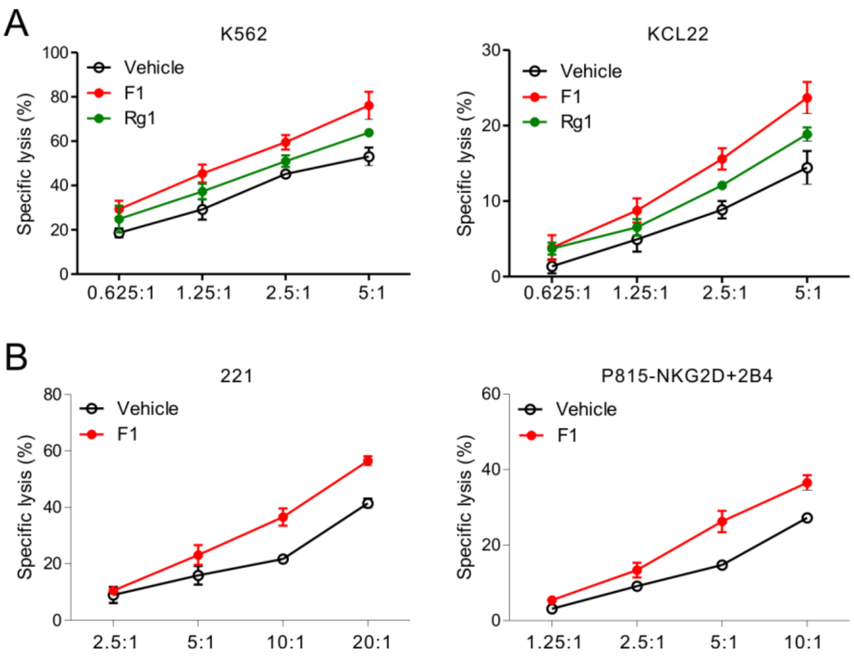

Figure S6. Ginsenoside F1 enhances cytotoxicity of primary NK cells and NK cell line.

(A) Lysis of target cells (K562 or KCL-22) by expanded primary NK cells after treatment with the indicated ginsenosides (10 μM) or vehicle only for 20 h at the indicated effector to target (E:T) cell ratio. Cytotoxicity against target cells was measured after 2 h with the Europium assay. Error bars represent the s.d.

(B) Lysis of target cells (721.221 or P815) by NKL cell line after treatment with G-F1 (10 μM) or vehicle only for 20 h at the indicated effector to target (E:T) cell ratio. Cytotoxicity against target cells including P815 cells preincubated with monoclonal antibodies specific for NKG2D and 2B4 was measured after 2 h using the Europium assay. Error bars represent the s.d. Data are representative of at least three independent experiments.

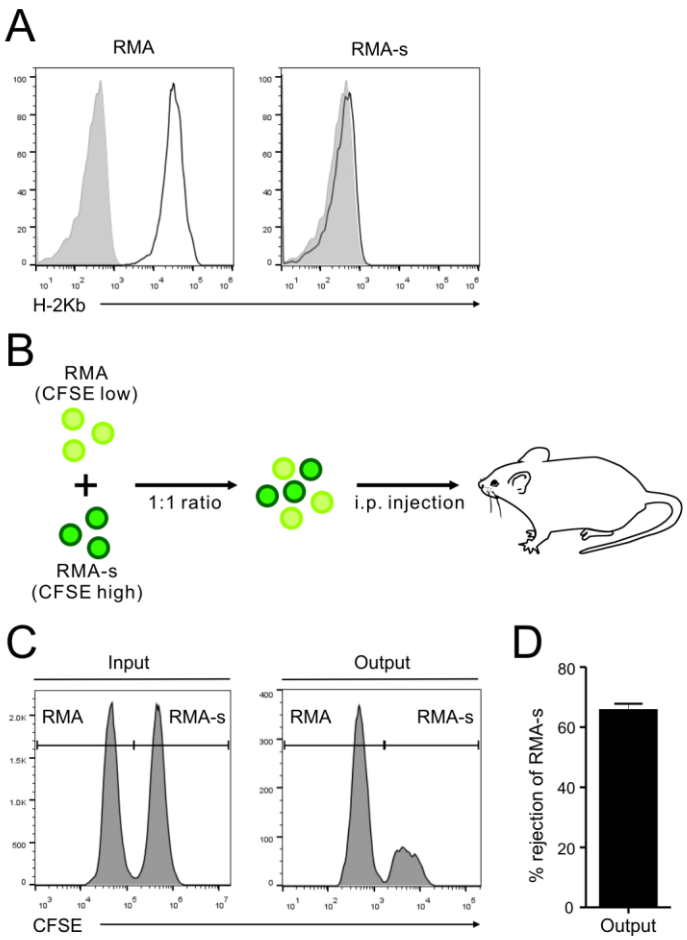

Figure S7. Syngeneic lymphoma rejection assay *in vivo.*

(A) Representative flow cytometry profile showing the expression of the MHC class-I (H-2Kb) on RMA and RMA-s cells (solid line). Isotype control staining is shown as a shaded histogram. Data are representative of three independent experiments.

(B) *In vivo* lymphoma rejection protocol. Lymphoma cells expressing MHC class-I (RMA) were labeled with a low CFSE concentration, whereas those with defective expression of MHC class-I (RMA-s) were labeled with a high CFSE concentration. A 1:1 cell mix was then injected i.p. into C57BL/6 mice, and the rejection of NK cell-sensitive RMA-s relative to NK cell-resistant RMA cells in the peritoneal cavity was measured using flow cytometry.

(C, D) Flow cytometry of CFSE-stained RMA and RMA-s cells injected at a ratio of 1:1 (Input)

into mice (n = 3), followed by analysis of CFSE in cells recovered from peritoneal cavity of recipient mice 12 h later (Output) to assess NK cell killing activity *in vivo*. Representative result (C) and summary graph of bar chart (D) showing percentage of rejected RMA-s cells.

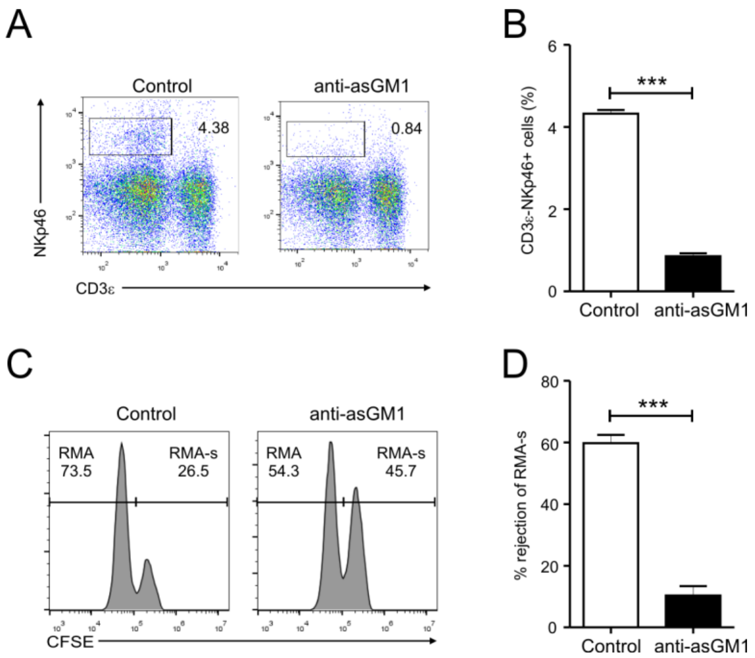

Figure S8. *In vivo* rejection of RMA-s cells are NK cell-dependent.

(A, B) C57BL/6 mice (n = 4 per group) were treated with i.p. injection of anti-asialo-GM1 (10 μL) or control serum for three consecutive days before an i.p. injection of CFSE-stained RMA (low CFSE concentration) and RMA-s cells (high CFSE concentration) at a ratio of 1:1. After 12 h post challenge of RMA:RMA-s cells, mice were sacrificed and the frequencies of NK cells (CD3ε-NKp46+) in the spleen of each group are shown as representative flow cytometry profile (A) and summary graph of statistical bar charts (B).

(C, D) NK cell-sensitive RMA-s cells relative to NK cell-resistant RMA cells remained in the peritoneal cavity of the same mice as above were recovered and assessed using flow cytometry. Representative result (C) and summary graph of statistical bar charts (D) showing percentage of rejected RMA-s cells. Values represent means ± s.e.m. ***$p < 0.001$ by Student's t-test.

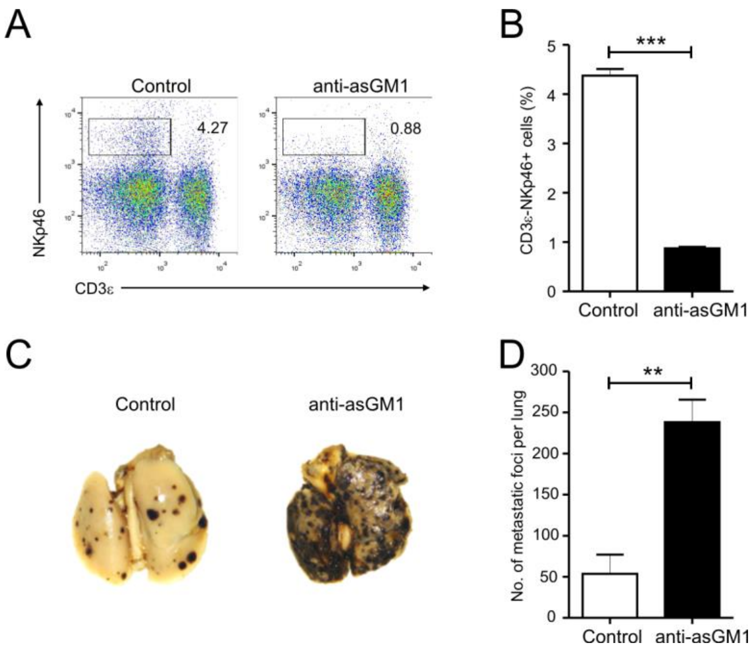

Figure S9. Pulmonary metastases of B16F10 cells are NK cell-dependent.

(A, B) C57BL/6 mice (n = 4 per group) were treated with i.p. injection of anti-asialo-GM1 (10 μL) or control serum before an i.v. injection of 2×10^5 B16F10 cells and twice a week treatment thereafter. After 14 day of tumor implantation, mice were sacrificed and the frequencies of NK cells (CD3ε-NKp46+) in the spleen of each group are shown as representative flow cytometry profile (A) and summary graph of statistical bar charts (B). Data are expressed as means ± s.e.m. (C, D) Lungs from the same mice as above were obtained and the number of metastatic tumor colonies in the lung was counted. Representative lung image (C) and summary graph of statistical bar charts (D) showing pulmonary metastases of B16F10 cells. Values represent means ± s.e.m. ***p* < 0.01; ****p* < 0.001 by Student's *t*-test.

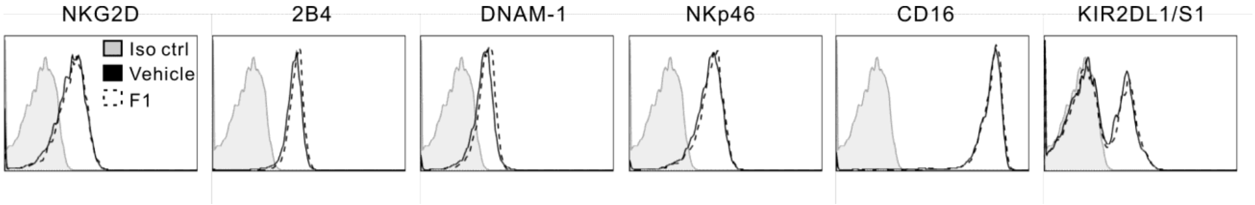

Figure S10. Effect of ginsenoside F1 on the expression of NK cell activating receptors. Representative flow cytometry profile showing the expression of the NKG2D, 2B4, DNAM-1, NKp46, CD16 and KIR2DL1/S1 receptors on gated CD3-CD56+ NK cells after incubation with G-F1 (10 μM; dashed line) or with vehicle only (solid line) for 20 h. Isotype control staining is shown as the shaded histogram. Data are representative of at least three independent experiments.
